# The gut microbiome associated with LGI1‐antibody encephalitis

**DOI:** 10.1111/epi.18556

**Published:** 2025-08-06

**Authors:** Edmund Gilbert, Sophie Binks, Valentina Damato, Christopher Uy, Paula Colmenero, Mark Kelly, Mohamed Ibrahim Khalil, Marcus O'Brien, Marcus J Claesson, John F. Cryan, Norman Delanty, Sarosh R. Irani, Gianpiero L. Cavalleri

**Affiliations:** ^1^ School of Pharmacy and Biomolecular Sciences Royal College of Surgeons in Ireland Dublin Ireland; ^2^ FutureNeuro SFI Research Centre Royal College of Surgeons in Ireland Dublin Ireland; ^3^ Oxford Autoimmune Neurology Group, Nuffield Department of Clinical Neurosciences University of Oxford, Level 3, West Wing, John Radcliffe Hospital Oxford UK; ^4^ Department of Neurology John Radcliffe Hospital Oxford UK; ^5^ Department of Neurosciences Drugs and Child Health University of Florence Florence Italy; ^6^ Oxford Centre for Microbiome Studies Kennedy Institute, University of Oxford Oxford UK; ^7^ SeqBiome Ltd., Moorepark Food Research Centre Cork Ireland; ^8^ APC Microbiome Ireland University College Cork Cork Ireland; ^9^ School of Microbiology, University College Cork Cork Ireland; ^10^ Department of Anatomy and Neuroscience University College Cork Cork Ireland; ^11^ Departments of Neurology and Neurosciences, Mayo Clinic Florida USA

**Keywords:** gut microbiome shotgun sequencing, HLA association, LGI1‐Ab encephalitis, short‐chain fatty acid depletion

## Abstract

**Objective:**

Autoimmune encephalitis is a cause of brain inflammation characterized by auto‐antibodies, which target cell surface neuronal proteins and lead to neuronal dysfunction. The most common form is associated with auto‐antibodies to leucine‐rich glioma‐inactivated 1 (LGI1) protein, the presentation of which includes frequent focal seizures. The exact cause of these auto‐antibodies remains unknown, but established predispositions include overrepresented human leukocyte antigen (*HLA*) alleles. Yet, these *HLA* alleles are themselves common in the healthy ancestry‐matched population. One potential etiological hypothesis is that an environmental trigger, such as the gut microbiome, interacts with a genetically predisposed individual.

**Methods:**

To investigate this, we studied 42 patients with LGI1‐antibody encephalitis (LGI1‐Ab‐E) and 27 familial/environmentally matched controls, and performed metagenomic shotgun sequencing, to describe the compositional and functional differences in the gut microbiome.

**Results:**

We observed that LGI1‐Ab‐E gut microbiomes exhibited a significant reduction in the ratio of *Firmicutes* (or Bacillota) and *Bacteroidetes* phyla, which is associated with the dosage of *HLA* susceptibility allele count in patients with LGI1‐Ab‐E. Furthermore, we identified differences in functional gene profiles in the gut microbiome that led to a reduction of neuroinflammatory protective short‐chain fatty acids (SCFAs) in LGI1‐Ab‐E patients.

**Significance:**

Taken together, our results suggest that a compositional shift in the gut microbiome of LGI1‐Ab‐E associates with a neuroinflammatory state, possibly through the reduction of SCFA production. Our study highlights the potential of the gut microbiome to explain some of the complex condition and unravel etiological questions. Validation studies with greater sample sizes are recommended.

**Key points**

- No single bacterial species was significantly enriched or depleted in LGI1‐Ab limbic encephalitis cases, relative to controls
- We observed a significant reduction in the ratio of Firmicutes to Bacteroidetes (F/B) microbiota in LGI1‐Ab LE cases, relative to controls
- HLA‐DRB1*07:01 allele dosage in LE cases is potentially associated with a modest reduction of the F/B ratio
- In combination, the results suggest that a compositional shift in the gut microbiome of LGI1‐Ab‐E associates with a neuroinflammatory state, possibly through the reduction of short‐chain fatty acid production.

## 1 INTRODUCTION

Autoimmune encephalitis (AE) is characterized by the presence of autoantibodies with pathogenic potential that target cell surface neuronal proteins.^1^, ^2^, ^3^ One of the most common autoantibodies detected in AE binds leucine‐rich glioma‐inactivated 1 (LGI1) protein. Many of these patients typically have very frequent, focal seizures in addition to altered mood, personality change, and cognitive impairment.^4^ This semiology of these seizures can be pathognomonic, as is well‐established for patients with faciobrachial dystonic seizures in association with LGI1antibodies.^5^, ^6^, ^7^ In addition, other unusual semiologies in LGI1‐antibody encephalitis (LGI1‐Ab‐E) include thermal, piloerection, autonomic, and emotional events.^8^, ^9^ These clinical observations imply that autoantibodies mediate forms of seizures that are likely to have characteristic, and sometimes novel, underlying molecular mechanisms.

In addition to distinctive clinical features, these AE patients are often resistant to antiseizure medication (ASM) resistant, but show beneficial responses to immunotherapies, including corticosteroids, plasma exchange, and intravenous immunoglobulins.^5^, ^6^, ^10^, ^11^ Yet, despite immunotherapies and ASMs, more than 70% of patients are left with long‐term deficits, which impair quality of life.^5^, ^12^, ^13^ In addition, around 40% of people treated with immunotherapies experience adverse drug effects,^5^, ^6^ and in the case of LGI1‐Ab‐E, up to 20% develop chronic epilepsy.^14^ Hence there is an unmet need for personalized immunotherapies that could be safe and effective in the long term.

Despite its potential to explain causation and address therapeutic needs, the etiology of these diseases is poorly understood. Recent works have demonstrated that more than 90% of patients with LGI1 antibodies carry specific class‐II human leukocyte antigen (*HLA*) alleles, namely HLA‐DRB1*07:01 (odds ratio [OR]: 26.4, 95% confidence interval [CI]: 8.5–81.5,^15^ OR: 85.5, 95% CI: 10.4–699.6^16^). A striking feature of this genetic finding is the relatively common underlying population frequencies of the *HLA* risk alleles. The HLA‐DRB1*07:01 allele, for example, is also carried by 27% of healthy European‐ancestry populations^15^, ^17^ (~12% globally^18^). Although these strong HLA Class II associations clearly implicate T cells in disease pathogenesis, their high general population frequencies only partially explain disease causation. Hence, we examined potential contributory environmental triggers.

The gut microbiome elegantly provides an interface to explain such an environmental trigger. Indeed, in a variety of autoimmune central nervous system diseases, there is growing evidence for an etiological link between the gut microbiome and the brain, principally via innate immunity and T cells.^19^, ^20^, ^21^ In several autoimmune diseases, skews in gut microbial populations have been discovered that may predispose to the illness.^19^, ^22^, ^23^, ^24^ Furthermore, in autoantigen‐specific illnesses, a distinctive microbial signature may provide a similarly elegant link to the LGI1 autoantigen through molecular mimicry.

Here we investigated the gut microbiome in patients with LGI1‐Ab‐E. We also collected stool and salivary DNA from matched but unaffected relatives, close family members, or friends. We sought to (1) determine if species abundance or diversity in the gut microbiome was significantly different between healthy and affected family members, (2) characterize potential functional differences in these microbiotas, and finally (3) investigate the evidence of LGI1 sequence homologues within the gut microbiota of LGI1‐Ab‐E patients.

## 2 RESULTS

### 2.1 Recruitment and cohort

We recruited 42 patients with a clinically and serologically confirmed diagnosis of LGI1‐Ab‐E. In parallel, we recruited matched healthy controls (HCs, *n* = 27) through close family members who were typically a domestic partner (see Table 1). Patient antibody status was ascertained by cell‐based assay on serum samples, as described previously.^6^ We isolated both genomic DNA from blood and gut microbiome DNA from stool (see Methods). All cases and HCs were consistent with British‐ or Irish‐like ancestry in comparison to ancestry controls (Figure S1). *HLA* imputation confirmed an enrichment of the HLA DRB*07:01 allelotype in LGI1 patients (Table 1) compared to HCs. Immunotherapy (IT) regimens at the time of sampling included corticosteroids (*n* = 28, Table 1) and, of the others, a history of intravenous immunoglobulins (*n* = 10) and plasma exchange (*n* = 5). Most cases (*n* = 30) were also exposed to ASMs.

**TABLE 1:** Study cohort characteristics.

	Sample type	
	LGI1‐Ab‐E	Healthy controls
No. of patients	42	27
Control type	NA	
Spouse/partner		15/27 (56%)
Sibling		1/27 (4%)
Child		1/27 (4%)
Other		3/27 (11%)
Unknown		6/27 (22%)
Mean (median) age at sampling^a^	66 (66)	56 (58)
Sex, female, %^b^	8/42 (19%)	23/27 (85%)
Time from symptom onset, months		NA
Mean (median) [range] missing	41 (23) [.5–126] 3	
Type^c^		
Chronic	25	
Acute	14	
Clinical syndrome, %		NA
Limbic encephalitis	34/42 (81%)	
Morvan's syndrome	0/42 (0%)	
Neuromyotonia/pain	4/42 (10%)	
NA	2/42 (5%)	
Seizure symptoms, %		NA
Isolated seizures	2/42 (5%)	
Faciobrachial dystonic seizures	22/42 (52%)	
Any seizures^d^	37/42 (88%)	
Medications at time of sampling, %^e^		NA
Antiseizure		
Carbamazepine	2/42 (5%)	
Clobazam/clonazepam/lorazepam	2/42 (5%)	
Gabapentin/pregabalin	6/42 (14%)	
Lacosamide	1/42 (2%)	
Lamotrigine	5/42 (12%)	
Levetiracetam	16/42 (38%)	
Phenytoin	2/42 (5%)	
Valproate	6/42 (14%)	
None	10/42 (24%)	
NA	3/42 (7%)	
Immunotherapy		
Azathioprine	1/42 (2%)	
Corticosteroids	23/42 (55%)	
Mycophenolate mofetil	2/42 (5%)	
None	15/42 (36%)	
NA	3/42 (7%)	
HLA status, %^f^		
DRB1*07:01 carrier	37/42 (88%)	11/27 (41%)
DRB1*07:01 homozygote	2/42 (5%)	1/27 (4%)
DRB1*07:01 negative	3/42 (7%)	15/27 (41%)

*Note*: Shown are the cohort descriptors for 43 LGI1‐antibody encephalitis (LGI1‐Ab‐E) cases and 27 matched healthy controls. Cohort is characterized by genetic sex (sex), whether an individual is inferred to be a HLA DRB*07:01 carrier through imputation (HLA DRB status), and what relationship the health control has with matched case (HC type). For relationships not corresponding to “spousal,” “sibling,” or “child,” HCs were classified as “other,” and if no relationship data were provided, HCs were classified as “unknown.” Where “NA” is provided, this corresponds to case characteristics when describing healthy controls, or control characteristics when describing cases.

^a^ No age at sampling for 2 patients and 19 HCs.

^b^ No sex status for one patient.

^c^ Cases were classified as “acute” if their time since symptom onset was <18 months, otherwise they were classed as “chronic.”

^d^ No confirmed seizure data for two patients.

^e^ Seven cases were taking multiple ASMs.

^f^ *HLA* imputation performed for cases and HCs based on SNP genotypes.

### 2.2 Gut microbiome composition

We first compared the composition of the gut microbiome in cases vs controls. We generated gut microbiome genomic profiles from paired‐end metagenomic shotgun sequencing (see Methods and Figures S2 and S3) and investigated composition changes with differential abundance and alpha and beta diversity analysis. Shotgun sequencing generated an average of 8.7 million paired end reads per sample, where 84% of these reads passed the adapter trimming and quality filtering stage. An average of 91% of reads passed quality control and were classified using DNA species abundance estimators: *Kraken2* and *Bracken*.

**FIGURE 1:**
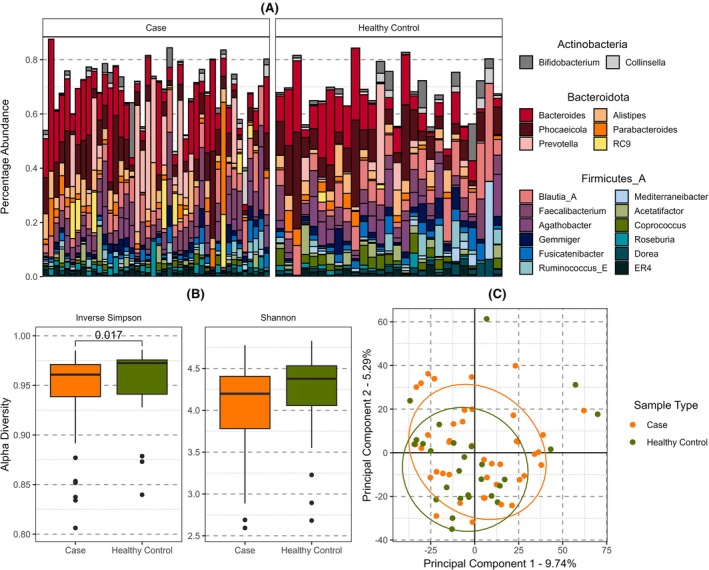
Comparison of the gut microbiome in LGI1‐antibody encephalitis cases and healthy controls (HCs). (A) Bar chart of the 20 most abundant genera, grouped by phylum and separating cases (left) and HC samples (right) in separate panels. Each vertical bar represents the compositional microbiome profile of an individual. Firmicutes_A and similar names are placeholder taxa that have been classified as such in the NCBI database but do not meet the clustering requirements for Genome Taxonomy Database. Unused space represents other species not in the top 20 most abundant. (B) The distribution of alpha diversity values between cases (orange) and HCs (green) using two measures, the Inverse Simpson and Shannon, to retain comparative direction of effect between the two. Pairwise *p*‐values shown are Wilcoxon test *p*‐values significant after Holm correction for multiple testing (Shannon not significant). (C) Principal component analysis of beta‐diversity estimates between cases and HCs. Ellipses shown are 80% confidence intervals assuming a multivariate t‐distribution.

The general compositional profiles of cases and HCs were consistent with the expected common human gut microbiome species such as *Bacteroides* or *Faecalibacterium* ^25^ (Figure 1A). We tested for differences in the abundance of individual species and identified no statistically significant differences that survived correction for multiple testing (Table 2). Given the rarity of this disease and the fine‐grained nature of individual species composition, the 18 most differentially abundant species are reported with uncorrected *p*‐values of < .05 (Table 2, Data S2). Several of the species nominally enriched in LGI1‐Ab‐E cases are also associated with the human oral and small intestinal microbiome (e.g., *Streptococcus parasanguinis*, *Streptococcus salivarius*, *Bifidobacterium dentium* (see Data S2), and various *Veillonella*).

**TABLE 2:** Differential gut microbiome species between LGI1‐Ab‐E cases and healthy controls.

Name	Level	Effect	Uncorrected	Corrected
			*p*‐value	*p*‐value
Streptococcus parasanguinis B	Species	.334	.0055	.7148
Haemophilus D parainfluenzae	Species	.321	.0102	.7407
Streptococcus salivarius	Species	.296	.0109	.7681
Veillonella atypica	Species	.470	.0112	.7370
Haemophilus D parainfluenzae K	Species	.435	.0145	.7657
NK3B98 sp900545815	Species	−.309	.0164	.8002
*Streptococcus sp001556435*	Species	.350	.0201	.8068
*GCA‐900066995 sp900291955*	Species	−.287	.0214	.8517
*Veillonella dispar A*	Species	.397	.0271	.7493
*UMGS1071 sp900542375*	Species	−.326	.0283	.8271
*Faecalibacterium prausnitzii A*	Species	.247	.0304	.8869
*Bifidobacterium dentium*	Species	.356	.0350	.8323
*Haemophilus D sp001815355*	Species	.304	.0401	.8169
*Streptococcus vestibularis*	Species	.380	.0448	.8340
*COE1 sp001916965*	Species	.282	.0466	.9068
*Alistipes A sp900240235*	Species	−.375	.0466	.8751
*Coprococcus sp900066115*	Species	−.268	.0479	.9097

*Note*: Shown are the 18 differentiated species that have significant, uncorrected for multiple testing, *p*‐values using the Kruskal–Wallis test using 512 Monte‐Carlo instances drawn from the Dirichlet distribution. Corrected *p*‐values are shown using the Benjamini–Hochberg correction method. A positive value indicates an enrichment in cases.

**FIGURE 2:**
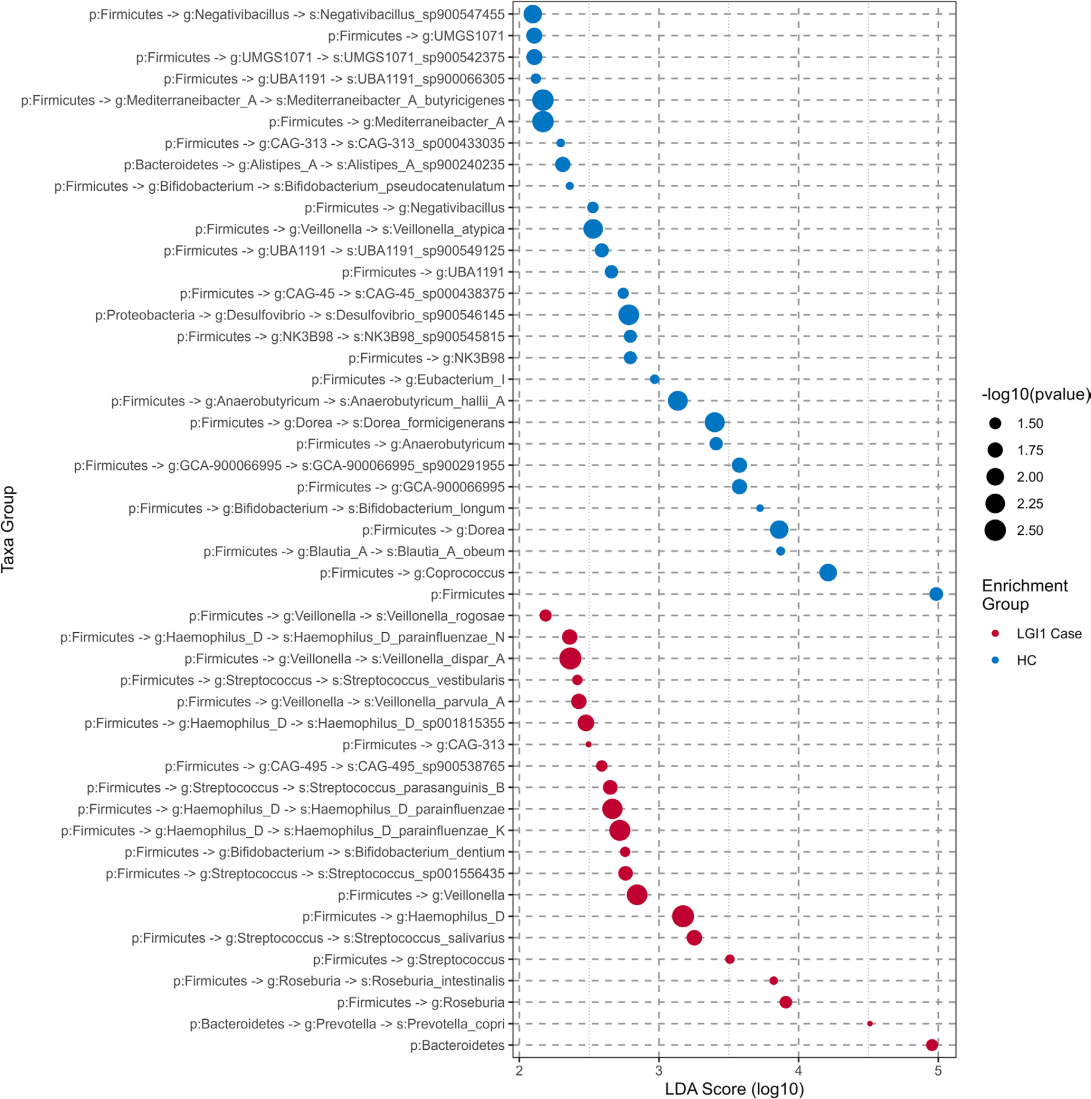
Differential enrichment of microbiome taxa in LGI1‐antibody encephalitis cases and healthy controls (HCs). Taxa significantly (adjusted *p*‐value <.05) enriched in either HCs or cases. Points are color coded according to the sample type in which the taxon is enriched, and the log_10_(LDA) score (effect size) is shown along the x‐axis. Labels along the y‐axis are in the format of “p:” to indicate phylum, “g:” to indicate genus, and “s:” to indicate species with “‐>” joining parent and child taxa.

To complement and extend this fine‐grained species analysis, we simultaneously investigated phyla‐, genera‐, and species‐level enrichment between cases and controls using linear discriminant analysis (LDA) (Figure 2, Data S3). Supportive of previous species‐level analysis, our LDA results also provide evidence of human oral and small intestinal species of the *Veillonella* and *Streptococcus* genera enriched in cases. As corticosteroids may impact saliva‐biome quality,^26^ we used LDA to compare healthy controls and cases with and without a history of corticosteroid treatment. We observed that some enrichments of oral or small intestinal microbiota were associated with corticosteroid use (Figure S4). This did affect key differentially abundant species in Table 2 and Figure 2. In addition, our cases were enriched for the *Enterococcus* and *Haemophilus D* genera, with depletion of the *Dorea* and *Coprococcus* genera, and the Firmicutes phylum (recently renamed *Bacillota* ^27^, ^28^). Beyond these depletions in HCs, we also observe a depletion specifically of *Bifidobacterium longum* in cases (Figure 2). Finally, as ASMs may also impact gut microbiome composition,^29^, ^30^, ^31^ we used LDA to compare ASM use vs non‐ASM use and observed very few differences (Figure S5).

From the case vs control LDA, the differential balance of the *Firmicutes* and *Bacteroidetes* phyla in LGI1‐Ab‐E cases and controls was of interest. Although changes in the *Firmicutes/Bacteroidetes* (F/B) ratio has been implicated in some human health–related traits,^32^, ^33^, ^34^ this remains debatable.^32^ Nevertheless, we investigated such large‐scale changes in the context of our modest sample size. As expected^33^ these two phyla account for the majority of phyla represented in the analysis: (F) 52.0% + (B) 40.0% (92.0% – Cases) and (F) 61.7% + (B) 30.4% (92.1% – HC). With analysis of the F/B ratio in HCs and the LGI1‐Ab‐E cases (Figure S6), we found the F/B ratio to be significantly lower in LGI1‐Ab cases relative to HCs (median F/B ratio in cases: 1.13, HCs: 2.28, Wilcoxon two‐sided *p*‐value: .030). Considering this F/B ratio difference between cases and controls we explored whether the dosage of HLA‐DRB1*07:01 is associated with this F/B compositional difference. As expected, we found an enrichment of HLA‐DRB1*07:01 heterozygotes in *LGI1*‐Ab‐E cases vs controls (Hardy–Weinberg equilibrium [HWE] exact *p*‐value: 1.3 × 10^−6^) but not in controls (exact *p*‐value: 1) (Figure S7). We observed the F/B ratio to modestly decrease as a measure of HLA‐DRB1*07:01 dosage, although this correlation did not reach statistical significance (Spearman's rank correlation = −.25 (*p*‐value = .11). The same pattern was not observed in the HCs (*p*‐value = .66) (Figure S7). To further explore this observation, we fitted F/B ratio in a multivariate glm model, which tested HLA‐DRB1*07:01 allele dosage, case/control status, sex, and age. The only significant variable remaining was HLA‐DRB1*07:01 allele dosage (*p*‐value = .045, *β* = −2.04, −3.96 to −.12 [95% CI]). We noted an apparent outlier with an F/B ratio of >9, and we found that this was an influential outlier with a standardized residual of 3.71. Consequentially omitting this individual from the model improved the fit as measured by the Akaike Information Criteria (from 185.25 to 162), and the significance of HLA‐DBR1*07:01 allele dosage (*p*‐value = .0013, *β* = −2.53, −3.96 to −1.11 [95% CI]).

We next investigated within‐gut and between‐gut diversity. Testing within‐gut with Chao1, Simpson, and Shannon alpha diversity measures we found evidence of subtle differences between case and HC status—consistent with the observations from differential species analysis and LDA. We observed inverse Simpson alpha diversity to be significantly enriched in HC compared to LGI1‐Ab cases (Wilcox *p*‐value = .015), as well as Shannon (Wilcox *p*‐value = .045) (Figure 1B, Figure S8). Investigating between‐gut diversity we performed principal component analysis (PCA) of Aitchison distances of these abundance data but found no significant differences along these components between cases and controls (Figure 1C, Figure S9). We also observed significant differences between dietary variables and these principal components (PCs; Data S4), although the uneven sampling size or extremes in scale (such as vitamin most B12 consumption is “infrequent” or “daily”) leads us to caution against these dietary‐based observations.

### 2.3 Functional characteristics

We next sought to extend the suggestive findings of differences in case–control gut microbiome profiles, characterizing function through Kyoto Encyclopedia of Gene and Genomes (KEGG) modules and pathways^35^ using HUMAnN3^36^ (see Methods). We tested both high‐level KEGG modules and lower‐level pathways for differential abundance using *Aldex2*,^37^ using an uncorrected *p*‐value of .05 similar to our differential species analysis (Table 3, Data S5 and S6). We observed several KEGG modules to be significantly different in cases and controls at an uncorrected level. The strongest enrichment was in HCs and covered coenzyme A (CoA) biosynthesis and production of short‐chain fatty acids (SCFAs). Of the KEGG pathways, all but pentose and glucuronate interconversions (KEGG pathway map00040) were also enriched in HCs. In PCA of these KEGG pathway differences, we did not observe significant clustering of cases vs HCs on PCs 1−3 (Figure S10), or in PCA of KEGG module differences (Figure S11).

**TABLE 3:** Differential KEGG modules between LGI1‐Ab‐E cases and healthy controls.

	ID	Name	Effect	Uncorrected *p*‐value	Corrected *p*‐value
Module	M00120	Coenzyme A biosynthesis, pantothenate = > CoA	.482	.0063	.3831
	M00873	Fatty acid biosynthesis in mitochondria, animals	.350	.0247	.4917
	M00026	Histidine biosynthesis, PRPP = > histidine	.334	.0317	.5335
	M00530	Dissimilatory nitrate reduction, nitrate = > ammonia	−.283	.0319	.5351
	M00874	Fatty acid biosynthesis in mitochondria, fungi	.347	.0383	.5383
	M00046	Pyrimidine degradation, uracil = > beta‐alanine, thymine = > 3‐aminoisobutanoate	.285	.0384	.5551
	M00028	Ornithine biosynthesis, glutamate = > ornithine	.316	.0473	.5501
	M00159	V/A‐type ATPase, prokaryotes	.274	.0518	.5647
	M00033	Ectoine biosynthesis, aspartate = > ectoine	.329	.0522	.5704
	M00364	C10‐C20 isoprenoid biosynthesis, bacteria	.313	.0538	.5347
	M00118	Glutathione biosynthesis, glutamate = > glutathione	−.278	.0548	.5527
	M00015	Proline biosynthesis, glutamate = > proline	.308	.0693	.5989
	M00017	Methionine biosynthesis, apartate = > homoserine = > methionine	.284	.0850	.6090
	M00529	Denitrification, nitrate = > nitrogen	−.302	.0867	.5756
	M00375	Hydroxypropionate‐hydroxybutylate cycle	.215	.0930	.6125
Pathways	map00791	Atrazine degradation	−.491	.0023	.1715
	map00040	Pentose and glucuronate interconversions	−.437	.0059	.2691
	map00643	Styrene degradation	.386	.0074	.2700
	map03008	Ribosome biogenesis in eukaryotes	.357	.0134	.3392
	map05012	Parkinson disease	.376	.0154	.3812
	map04621	NOD‐like receptor signaling pathway	.344	.0219	.4346
	map04910	Insulin signaling pathway	.343	.0254	.4393
	map00121	Secondary bile acid biosynthesis	.354	.0337	.4678
	map00120	Primary bile acid biosynthesis	.345	.0422	.4861
	map05205	Proteoglycans in cancer	.301	.0450	.5420
	map05418	Fluid shear stress and atherosclerosis	.318	.0534	.5923
	map04217	Necroptosis	.280	.0573	.6001
	map05150	Staphylococcus aureus infection	−.290	.0675	.5974
	map00633	Nitrotoluene degradation	.223	.0785	.6910

*Note*: Shown are the differentiated KEGG modules, and the KEGG pathways that have significant, uncorrected for multiple testing, *p*‐values using the Kruskal–Wallis test using 512 Monte‐Carlo instances drawn from the Dirichlet distribution. Corrected *p*‐values are shown using the Benjamini–Hochberg correction method. A positive value indicates an enrichment in cases.

Finally, we tested the evidence that the gut microbiome of LGI1‐Ab‐E patients contains potential bacterial homologues of the human *LGI1*, searching the *RefSeq* database for *LGI1* homologues with *blastp* (see Methods for quality cutoffs). We identified 13 organisms to contain such homologues (Data S7); however, the majority potential homologues are annotated as “leucine‐rich” or “hypothetical,” and nearly all organisms in which these homologues are observed are typically found in marine environments. Mapping these potential homologues to the metagenomic data of our LGI1‐Ab‐E cases, we do not observe any hits and no evidence of their presence in the gut microbiome of LGI1‐Ab‐E cases.

## 3 DISCUSSION

This metagenomic analysis of the gut microbiome in 42 patients with LGI1‐Ab‐E, matched with 27 HCs with paired genomic data, suggests a potential compositional bias in the gut microbiome of AE. Together our results suggest subtle taxa compositional changes in cases with these differences potentially associated with HLA‐DRB1*07:01 dosage. Furthermore, this compositional change was associated with depletion of KEGG modules linked to SCFA generation in LGI1‐Ab‐E cases.

We primarily observed that the general gut microbiome of individuals with LGI1‐Ab‐E appeared largely consistent with a normal microbiome composition, with a high abundance of common microbiome species.^25^ We therefore infer that LGI1‐Ab‐E is not characterized by a large difference in microbiome composition driven by specific species. The most positive signal of differing microbiome composition between cases and controls is the enrichment of *B. longum* in HCs. Of interest, this species has been observed to exert beneficial health effects on the human gut,^38^ inflammation,^23^ and potentially reduce cytotoxic effects of certain ASMs.^29^

Instead, LDA highlights several taxa potentially enriched in either our cases or controls, with some of these taxa associated previously with traits relevant to LGI1‐Ab‐E. Several genera enriched in our cases have been associated with neurological conditions and thus allows us to place our results into the context of the interplay of the microbiome and neuroinflammation. *Streptococcus*, for example, has been found to be associated with focal epilepsy in children,^39^ where in a small sample (*n* = 10) the authors posit a neuroinflammatory model. Indeed, a mouse model of intestinal inflammation increases convulsant activity and ASM resistance.^40^ In addition to *Streptococcus*, the taxa *Enterococcus* ^41^ and *Roseburia* ^42^ have been found to be associated with intractable childhood or drug‐resistant epilepsy (respectively), and *Roseburia* in another study of LGI1‐Ab‐E,^43^ matching our case subtype. The *genera Coprococcus* and *Dorea* are both enriched in our HCs. These have been associated with drug‐resistant epilepsy,^42^ but *Coprococcus* is also noted for its anti‐inflammatory associations^33^ and is depleted in our cases. We also performed LDA to examine differences associated with therapeutic approaches, finding that some corticosteroid use, but far less so ASMs, can alter gut microbiome compositions—broadly consistent with previous observations.^26^ Together, our results would support larger, multi‐center studies of the role of the gut microbiome in epilepsy health care, from ASM use to disease etiology, and longitudinally from onset of AE to chronic, expanding the sample size and resolution of disease outcomes.

In addition to subtle genera or species differences, our primary observation is the reduction of F/B ratio in LGI1‐Ab‐E cases compared to HCs. This reduction in the F/B ratio appears to follow HLA DRB*07:01 allele dosage in multivariate analysis, although this association did not reach statistical significance when tested in a univariate correlation with this sample size. The frequency of HLA DRB*07:01 in our LGI1‐Ab cases (91% carriers) matches that observed in other LGI1‐Ab‐E cohorts.^16^, ^17^, ^44^ To our knowledge this is the first observed association potentially linking gut microbiome compositional changes to the major LGI1‐Ab‐E genetic risk locus. It is encouraging that a reduction of the F/B ratio and taxa diversity has been associated in another gut microbiome study of LGI1‐Ab‐E in a cohort of 15 patients of Han Chinese ancestry compared to 25 matched controls using 16S rRNA sequencing.^43^ Although the Chinese investigation did not consider *HLA* genotypes, together our studies show evidence that a subtle shift in taxa composition measured by the F/B ratio is associated with LGI1‐Ab‐E and is consistent with an inflammatory disease model.^43^ However, caution is warranted. For obesity, one of the first traits where the F/B ratio was suggested as a biomarker, results are frequently discrepent^32^ and that phyla encompasses a wide variety of taxa with differing functional attributes.^22^ Wider and further studies are warranted in neuroinflammatory and epilepsy traits, to investigate the veracity of our observations. It is encouraging that these F/B ratio findings appear to be complemented by our functional characteristics results. We find three KEGG modules that are nominally depleted in cases and are particularly interesting in the context of neuroinflammatory epilepsy. Microbial CoA is a key source of SCFAs in the gut,^24^ where they are protective for neuroinflammation^43^ and seizure^45^ and epilepsy in general.^46^ Ornithine (Data S5) acts as a precursor to CoA in its acetyl‐CoA form^47^ and histidine acts as a precursor to ornithine.^48^ Taken holistically with the enrichment of species such as *B. longum* in HCs, an association between a specific HLA class II genotype suggests a model whereby compositional changes in the gut microbiome are associated with potential functional shifts linked to a loss of neuroprotective SCFAs.

The primary limitation of this study was cohort size. LGI1‐Ab‐E is a rare disease that can impact cognitive function, and despite recruitment across the UK and Ireland, only 43 LGI1‐Ab‐E cases were available for analysis. Indeed, initial recruitment also sampled four CASPR2‐Ab‐E cases, matching literature reports of the predominance of LGI1‐Ab‐E cases compared to CASPR2‐Ab‐E.^49^ The low CASPR2‐Ab‐E sample size prevented any meaningful analysis of this case subtype. In addition, we have studied cases of more chronic presentation rather than sampling exclusively at the acute stage of presentation. A chronic presentation may have a composition to the microbiome that is different from that of cases with an acute onset. We expect that this sample size constraint and the low ratio of cases to healthy controls impacted our ability to detect more subtle differences between LGI1‐Ab‐E cases and controls, thereby limiting the statistical power of sub‐group analysis. Combining with other AE cohorts of a similar dietary and pharmacological background may provide novel insights, although future care will need to be taken in harmonizing gut microbiome sequence generation methods to ensure that inter‐study comparisons are possible. Another limitation was the difference in the proportions of sexes in cases vs HCs, with a preponderance of HC females (85%, compared to 19% in LGI1‐Ab cases). We attribute this to our spousal‐ or close‐relative–based recruitment of HCs. Compositional changes in the microbiome have been associated with aging,^50^ and we lacked information on the ages of most of our HCs. However, we expect our cases and controls to be largely age‐matched (and background matched), as our HCs are predominantly spousal or sibling in relationship, and this is supported by the median age of 58 in 7 of 27 HCs compared to 66.

To conclude, our work suggests that LGI1‐Ab‐E is associated with specific compositional changes in the gut microbiome, shifting the balance between the predominant taxa *Firmicutes* and *Bacteroidetes* in favor of *Bacteroidetes*. This ratio change is associated with the significant HLA risk allelotype DRB*07:01 in cases only and matches previous studies of LGI1‐Ab‐E cases. This profile is also associated with a shift in functional microbial activity linked to reduced SCFA levels. Given our low sample size, reflective of this rare condition and its effect on statistical power, the exact effect that the microbiome plays in disease pathogenesis and/or progression remains unclear. However, our results show a role in a subtle compositional and functional change that is consistent with a neuroinflammatory model and case‐specific suggestive effect in the HLA DRB*07:01 allelotype. Further work with expanded sample sets and coordinated sequencing protocols appears to be the next step in elucidating the potential trigger for this rare and refractory neuroinflammatory condition.

## 4 METHODS

### 4.1 Ethics approval

This study was approved by ethics committees at the Royal College of Surgeons in Ireland (under protocol REC1631) and Oxford University (under protocol number 16/YH/0013). Ethics for this study was approved in the UK by Yorkshire & The Humber – Leeds East Research Ethics Committee (16/YH/0013). All patients gave written informed consent.

### 4.2 Recruitment

Eligible patients were ≥18 years of age with a clinical diagnosis of autoimmune encephalitis/epilepsy and positive serum LGI1 antibodies by a live cell‐based assay, as described previously.^6^ HCs were spouses, relatives, or friends attending clinic or subsequently recruited from the community, preferentially recruiting genetic relatives, or co‐habiting relatives. All participants gave written informed consent, or assent was given by next of kin. Saliva and stool samples were gathered in the home environment and posted via secure biokit. Stool was collected using an Omnigene Gut stool collection kit (DNA Genotek, OM‐200). Saliva was collected using Isohelix GeneFix saliva collection kits (Isohelix, GFX‐02). Samples were posted to the lab using an overnight tracked‐return service and stored at −80°C on arrival. At the time of sampling study participants also completed a questionnaire covering toileting routine, bowel habits, and dietary and medication intake (see Data S1 for this questionnaire).

### 4.3 DNA extraction

DNA was extracted from stool and saliva samples within 2 weeks of their arrival at the lab. Saliva extractions were performed using the Isohelix GeneFix Saliva‐Prep DNA Kit and stool extractions using the QIAamp PowerFecal Pro DNA Kit (Qiagen, 51 804). DNA quality was assessed using a Nanodrop One spectrophotometer.

### 4.4 SNP genotyping

Salivary DNA samples were genotyped on the Illumina OmniExpress chip at Edinburgh Genomics, according to manufacturer's instructions. Genomic ancestry analysis was performed using PCA from this single nucleotide polymorphism (SNP) genotype data. Case and HC genotypes were merged with Irish and British ancestry controls from the Irish DNA Atlas^51^ (*n* = 193), Irish Trinity Student^52^ (*n* = 2228), and People of the British Isles^53^ (*n* = 2039) using PLINK v1.9.^54^, ^55^ We filtered for samples and variants with a missingness <5%, and variants with a minor allele frequency <1% and a *p*‐value signifying deviation from Hardy‐Weinburg Equilibrium <1e^−9^. We performed PCA on a set of SNPs independent in terms of linkage disequilibrium, using the PLINK ‐‐indep‐pairwise command with a window of 1000, step size of 50, and linkage filtering threshold of .2.

Using these SNP genotypes, we performed *HLA* allele imputation using the Michigan Imputation Server,^56^ preparing genotypes for imputation using the McCarthy Group imputation preparation tools (https://www.chg.ox.ac.uk/~wrayner/tools/), and using the Four‐digit Multi‐ethnic HLA v2 (2022) *HLA* imputation panel reference.^57^ From the imputed genotypes we extracted the genotype status of the HLA DRB1*07:01 allele for all cases and HCs, which had an overall imputation *r* ^2^ value of .93.

### 4.5 Microbiome sequencing

Gut microbiome DNA samples were shipped to SeqBiome Ltd., Fermoy, County Cork, Ireland. Library construction and shotgun sequencing of autoimmune cases and controls were carried out at SeqBiome Ltd. using Nextera library preparation^58^ and Illumina NovaSeq sequencing (2 × 150 bp per sample, Illumina, Hayward, California, USA).

Gut microbiome sequence data were then processed initially with *FastQC*,^59^ where data quality was visually inspected. All samples were of generally good quality with minimal quality drop off at the end of sequences. *Trimmomatic* ^60^ was used for trimming and quality filtering using the following parameters: SLIDINGWINDOW:5:22, MINLENGTH:75. The data were then passed to *Kneaddata*, a pipeline incorporating *Trimmomatic*, *Bowtie 2*,^61^ and other elements designed for contaminant removal. Taxonomic assignment was then performed using *Kraken2* using a confidence of .1 and the *Kraken2* ^62^, ^63^ GTDB bacterial database.^64^ *Kraken2* is a taxonomic classification system using exact k‐mer matches to achieve high accuracy and fast classification speeds, using a customized version of the GTDB database, the results of which were analyzed using the R based phyloseq package.^65^ GTDB is a database that clusters available genomes based on Average Nucleotide Identity (ANI), and assigns taxonomy based on the National Center for Biotechnology Information (NCBI) classifications. If a sequence does not meet clustering requirements (95% ANI), but does not have a unique taxonomic classification, a suffix will be added to indicate this (e.g., *Escherichia coli* A). This allows for the most accurate taxonomic classifications, even in the case of organisms that have yet to be formally identified.

Pathway and gene family assignment was then performed using *humann3*.^36^ The standard Uniref90 annotation of gene families were collapsed into Kyoto Encyclopedia of Gene and Genomes (or KEGG)^35^ orthology annotation and further collapsed into KEGG module and then pathway annotation.

### 4.6 Comparative analysis

We used the phyloseq package^65^ in R^66^ v4.1.3 for comparative alpha diversity, beta diversity, and taxonomic analysis, using the package *ALDEx2*,^37^ to determine if any species were differentially abundant (at group level) based on a Kruskal–Wallis test, using 512 Monte‐Carlo instances drawn from the Dirichlet distribution. Initially corrected for Benjamini–Hochberg multiple testing, p‐values were all >.05. We therefore focused our discussion on the species with *p*‐values significant prior to multiple testing correction (<.05 prior to correction).

To account for the compositional structure of the Next Generation Sequencing (NGS)–generated data and to avoid the likelihood of generating spurious correlations, we first imputed the zeros in the abundance matrices using the count zero multiplicative replacement method (cmultRepl, method = “CZM”) implemented in the *Compositions* package and applied a centered log‐ratio (CLR) transformation using the *codaSeq.clr* function in the *CoDaSeq* package.

LDA effect size estimation was carried out using the *run_lefse()* function *microbiomeMarker* R package in Bioconductor on the species abundance matrix, selecting CPM normalization method, 100 bootstrap replicates, a Kruskal–Wallis and Wilcoxon *p*‐value cutoff of .05, and LDA effect size cutoff of 2. Note that immunotherapies beyond corticosteroids had extremely low sample size (Table 1), insufficient for a valid analysis. To test HLA‐DRB1*07:01 divergence from HWE expectations we utilized the R package HardyWeinberg and the function HWExact() to calculate an exact test for the HWE. To test correlations between HLA dosage and F/B ratio we used the R function cor.test(). We tested compositional changes measured by the F/B ratio between cases and controls using the wilcox. test() function in R. We tested the multivariate model using the glm() function in R, and detected outliers using the augment() function from the R package broom.

Alpha and beta diversity analysis was carried out using the *R* packages *vegan* and *phyloseq*, and statistical comparison was tested using Kruskal–Wallis tests. PCA was carried out using the *PCA* function in *R* using Aitchison distance matrix (CLR + Euclidean distances). To test the significance of groupings in PCA space, Permutational Multivariate Analysis of Variance (PERMANOVA) test was then performed using 999 permutations with a significance cutoff value of .05. Dispersion homogeneity was tested using the *betadisper* function from *vegan*.

### 4.7 Functional comparison

KEGG pathways and modules were assessed for differential abundance using *Aldex2* based on a Kruskal–Wallis test using 512 Monte‐Carlo instances drawn from the Dirichlet distribution. *p*‐values were initially corrected for multiple testing, and all were >.05. We therefore focused discussion on the pathways and modules with *p*‐values significant prior to multiple testing correction (<.05 prior to correction). KEGG pathways and KEGG modules were analyzed by computing PCAs using Aitchison distances. To test the significance of groupings, PERMANOVA tests were performed on these distances, using 999 permutations, with a *p*‐value cutoff of .05. Dispersion homogeneity was tested using the *betadisper* function from *vegan*.

To assess the presence of potential bacterial homologues of the human LGI1 protein, a *blastp* search of the LGI1 protein was run using a database of 64 695 bacterial genomes acquired from the *RefSeq* database from which proteomes were generated using *Prokka*.^67^ Potential homologues were then filtered from these results using cutoffs of >40% identity, >10% coverage, and an e‐value <1e‐10. These potential homologues were then mapped back to the metagenomic data using *blastx* with cutoffs of >80% identity, >80% coverage, and an e‐value of <1e‐6.

## AUTHOR CONTRIBUTIONS

Conceptualization and Funding Acquisition: G.L.C., S.R.I., N.D., J.F.C. and M.C. Data Curation: E.G., S.B., V.D., C.U., P.C., M.K., M.I.K., M.O.B., M.C. Patient recruitment and associated phenotyping: S.B., V.D., C.U., M.K., M.I.K., N.D., and S.R.I. Formal Analysis: E.G., S.B., and M.O.B. Investigation: E.G., and M.O.B. Methodology: E.G., M.O.B., and M.C. Project Administration: E.G., P.C., and G.L.C. Resources: P.C., and S.R.I. Software: M.O.B., and M.C. Supervision: P.C., J.F.C., N.D., S.R.I., and G.L.C. Visualization: E.G. Writing – Original Draft Preparation: E.G., S.B., S.R.I., and G.L.C. Writing – Review, editing and final approval: All authors.

## FUNDING INFORMATION

This work was funded by a grant from the Medical Research Charities Group, the Health Research Board, Ireland, and Epilepsy Ireland (grant code: MRCG‐2018‐005), and Science Foundation Ireland (SFI) under Grant Number 16/RC/3948 and co‐funded under the European Regional Development Fund and by FutureNeuro industry partners. A senior clinical fellowship (to SFI) from the Medical Research Council [MR/V007173/1], Wellcome Trust Fellowship [104079/Z/14/Z], the National Institute for Health Research (NIHR) Oxford Biomedical Research Centre (BRC). The views expressed are those of the author(s) and not necessarily those of the NHS, the NIHR, or the Department of Health.

## CONFLICT OF INTEREST STATEMENT

S.R.I. has received honoraria/research support from UCB, Immunovant, MedImmun, Roche, Janssen, Cerebral therapeutics, ADC therapeutics, Brain, CSL Behring, and ONO Pharma, and receives licensed royalties on patent application WO/2010/046716 entitled “Neurological Autoimmune Disorders”; and has filed two other patents entitled “Diagnostic method and therapy” (WO2019211633 and US‐2021‐0071249‐A1; PCT application WO202189788A1) and “Biomarkers” (PCT/GB2022/050614 and WO202189788A1).

## ETHICAL PUBLICATION POLICY

We confirm that we have read the Journal's position on issues involved in ethical publication and affirm that this report is consistent with those guidelines.

## OPEN ACCESS

For the purpose of Open Access, the author has applied a CC BY public copyright license to any Author Accepted Manuscript (AAM) version arising from this submission.

## Supporting information


Figure S1.

**Data S1.** Dietary and supplement questionnaire. A blank copy of the dietary and supplement questionnaire provided to study participants.

**Data S2.** Differential species. The identified differential species between LGI1‐antibody encephalitis cases and healthy controls, showing the effect size of difference and adjusted and unadjusted *p*‐values.

**Data S3.** Linear Discriminant Analysis taxa results. Results of the linear discriminant analysis of differential taxa between LGI1‐Ab‐E cases and healthy controls, showing in which group the enrichment was observed and the magnitude and adjusted *p*‐value.

**Data S4.** Dietary associations with beta‐PCs. The *p*‐values from PERMANOVA tests of association between beta‐diversity–derived principal components and dietary habits.

**Data S5.** All KEGG module results. The raw results of differential abundance of KEGG modules between LGI1‐antibody encephalitis cases and healthy controls, including size of effect and both corrected and uncorrected *p*‐values.

**Data S6.** All KEGG pathway results. The raw results of differential abundance of KEGG modules between LGI1‐antibody encephalitis cases and healthy controls, including size of effect and both corrected and uncorrected *p*‐values.

**Data S7.** Organism orthologues. The raw results from *blastp* search of the *LGI1* protein and mapped back to our metagenomic data with *blastx*. Shown are the homologue annotation, taxonomy of sequence, percentage identity with *LGI1*, coverage and mapping e‐value.

## Data Availability

The raw metagenomic sequence data will be made accessible on the European Genome‐Phenome Archive (EGA) upon publication, the details of which can be found at the project's github page: https://github.com/FutureNeuroIE/le‐microbiome‐2025.
