# Supplementary material for: The gut microbiome associated with LGI1‐antibody encephalitis
Source: Epilepsia. 2025 Aug 6;66(11):4411–24. doi: 10.1111/epi.18556 (PMC12661261; doi:10.1111/epi.18556)
Supplement: Supplementary file 1 — Figure S1. [file EPI-66-4411-s001.zip › AIE_Manuscript_Epilepsia_Supplementary_July_Clean.docx]

# Supplementary Materials


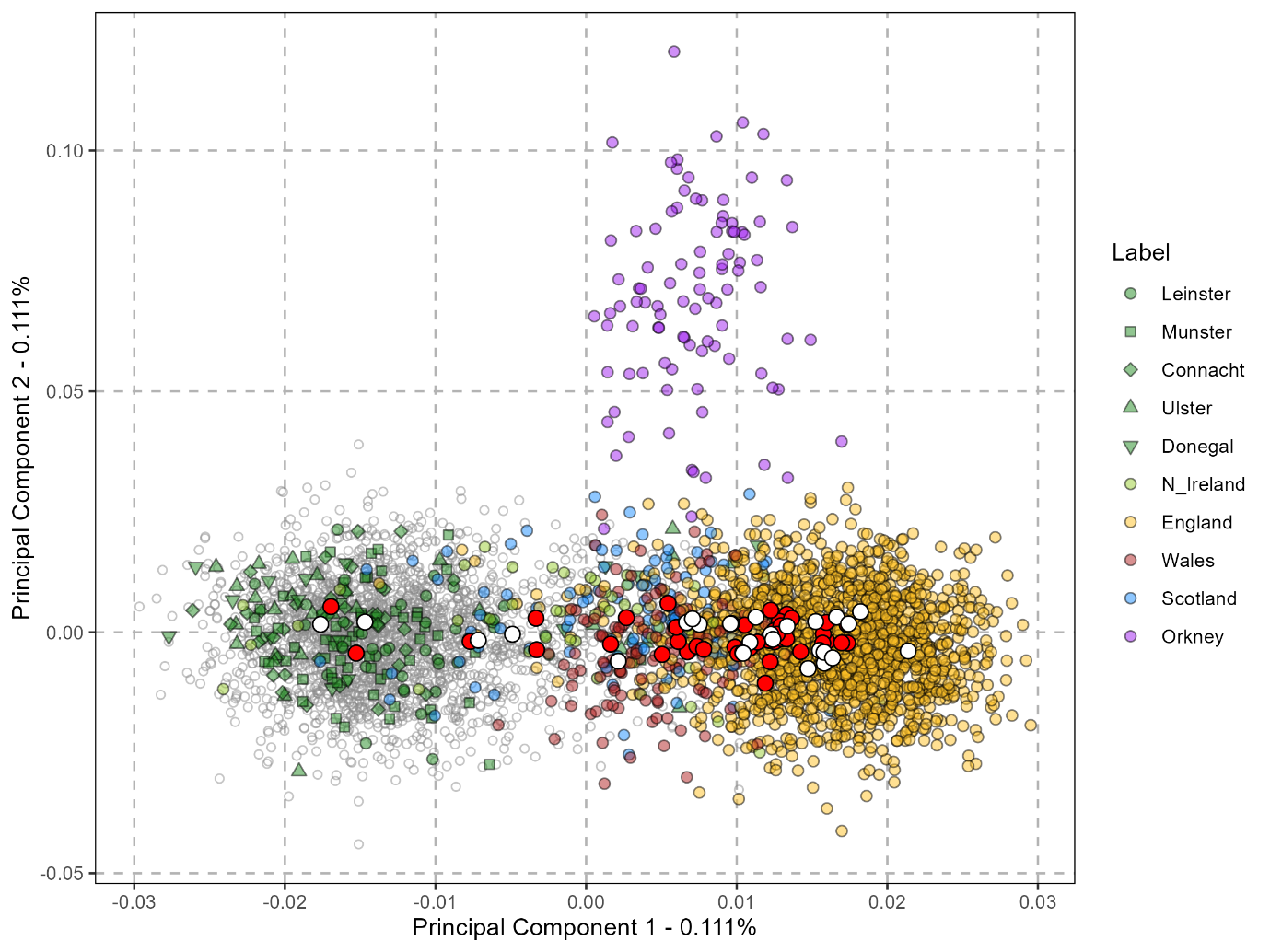


**Supplementary Figure 1 – Ancestry PCA of LGI1-Ab-E Cases and Controls**. The principal component decomposition of a genetic relationship matrix of 4,460 Irish and British ancestry controls and 42 cases and 27 health controls (HC). Points are colour and shape coded according to genetic ancestry group. Filled circles with red indicate the genetic position of a case and filled circles with white indicate the genetic position of a HC.


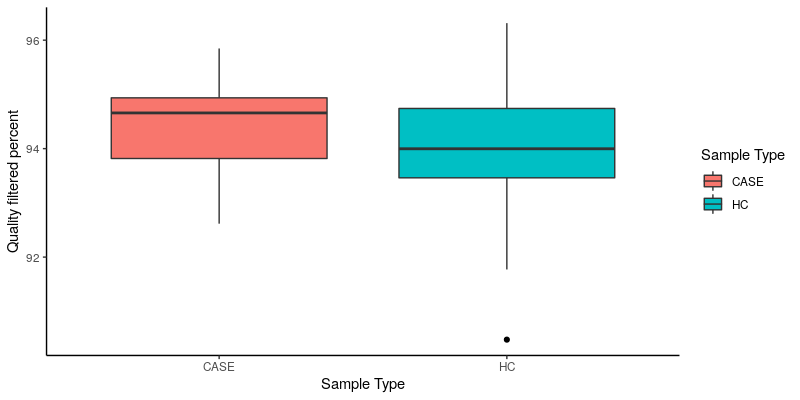


**Supplemental Figure 2 - Filtering quality of LGI1-Ab-E case and healthy control gut microbiome samples.** Boxplot representation of percentage of reads which pass the quality filtering step, coloured by sample type. Boxplots show the median value, with lower and upper hinges showing the 1st and 3rd quartiles. Whiskers show the largest value no further than 1.5 x the Interquartile Range (IQR) from that range. Data points beyond these whiskeys are plotted separately as black points.


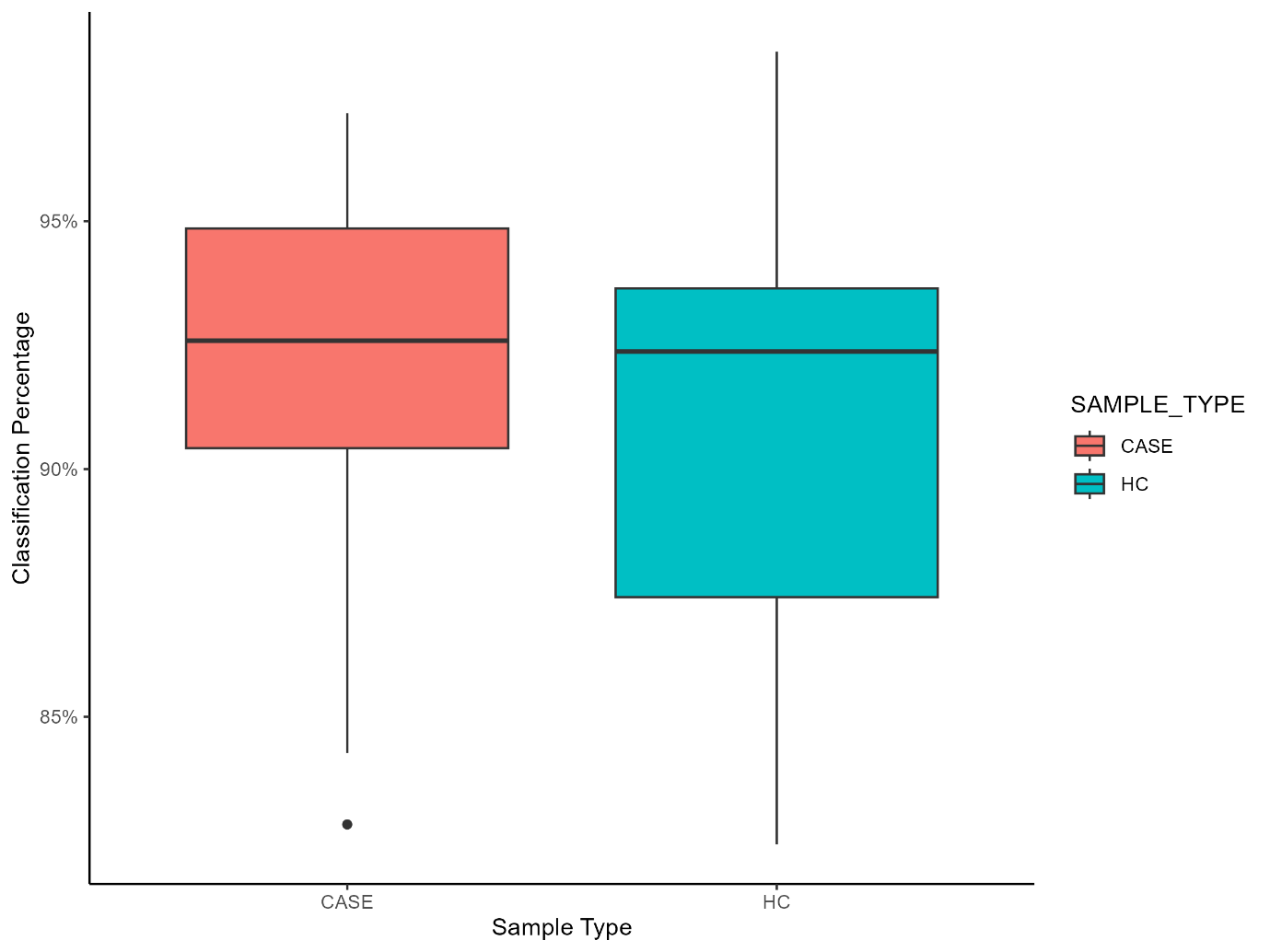


**Supplemental Figure 3 - Kraken2 percent classification of LGI1-Ab-E case and healthy control reads.** Boxplots show the median value, with lower and upper hinges showing the 1st and 3rd quartiles. Whiskers show the largest value no further than 1.5 x the Interquartile Range (IQR) from that range.


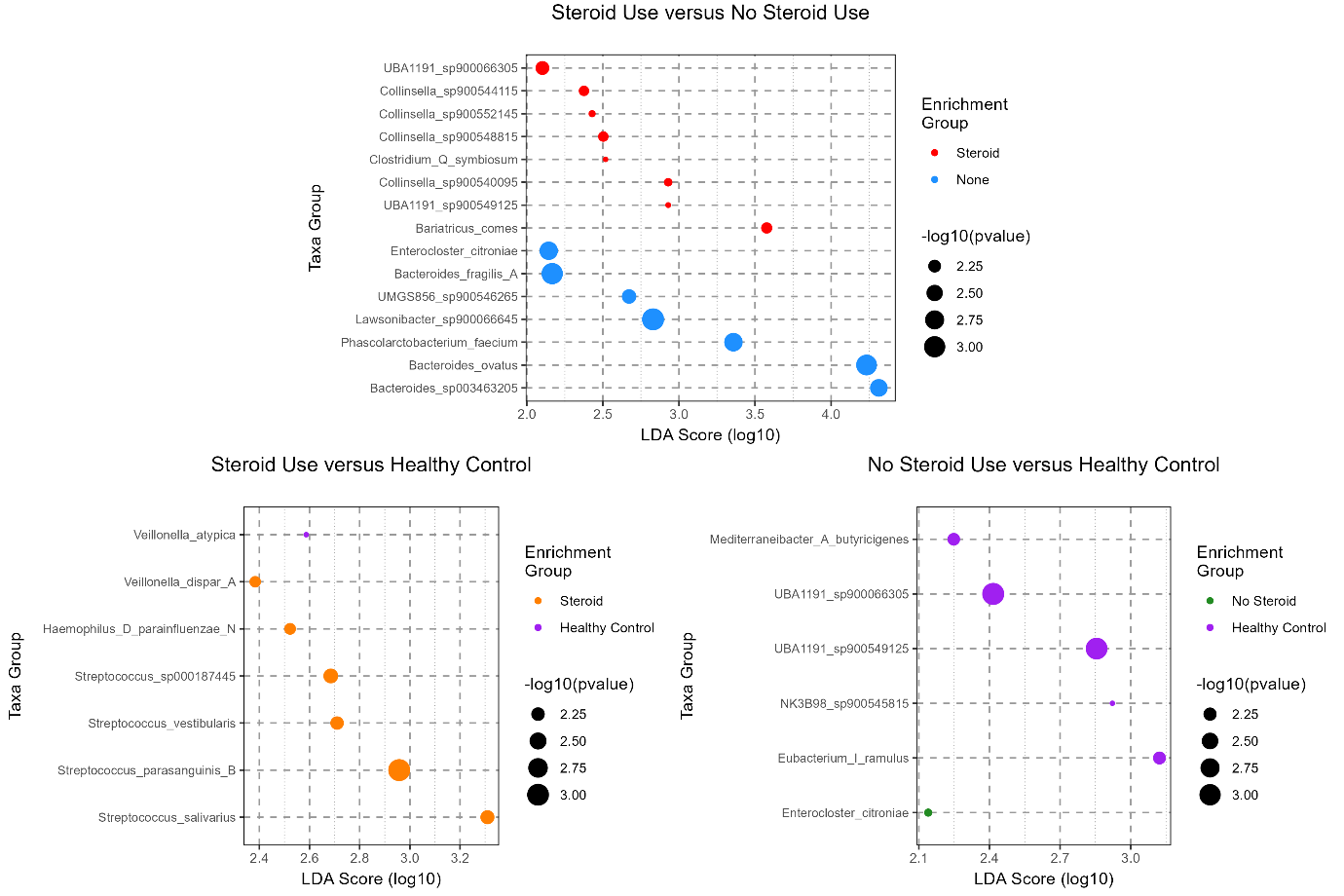


**Supplemental Figure 4 – Differential enrichment of microbiome taxa in LGI1-Ab-E cases stratified by steroid use history and healthy controls** - Taxa significantly (adjusted p-value < 0.05) enriched in either healthy controls (HC) or cases. Points are colour coded according to the sample type the taxon is enriched in, and the log_10_( LDA ) score (effect size) is shown along the x-axis. Labels along the y-axis are in the format of “p:” to indicate phylum, “g:” to indicate genus, and “s:” to indicate species with “->” joining parent and child taxa.


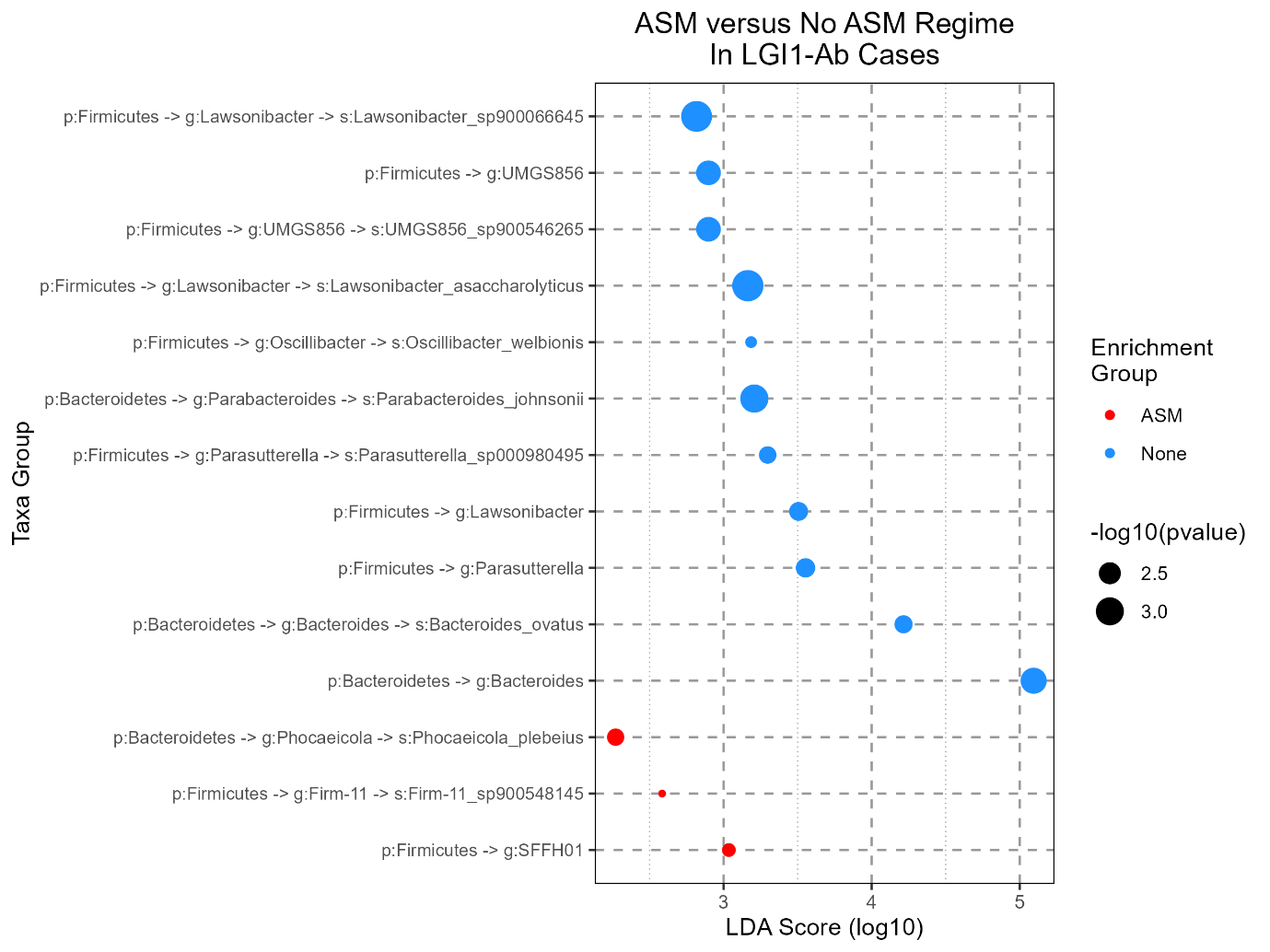


**Supplemental Figure 5 – Differential enrichment of microbiome taxa in LGI1-Ab-E cases stratified by anti-seizure medication (ASM) useU.** Taxa significantly (adjusted p-value < 0.05) enriched in either LGI1-Ab cases taking ASM at time of study (“ASM”) or those that did not (“None”). Points are colour coded according to the sample type the taxon is enriched in, and the log_10_( LDA ) score (effect size) is shown along the x-axis. Labels along the y-axis are in the format of “p:” to indicate phylum, “g:” to indicate genus, and “s:” to indicate species with “->” joining parent and child taxa.


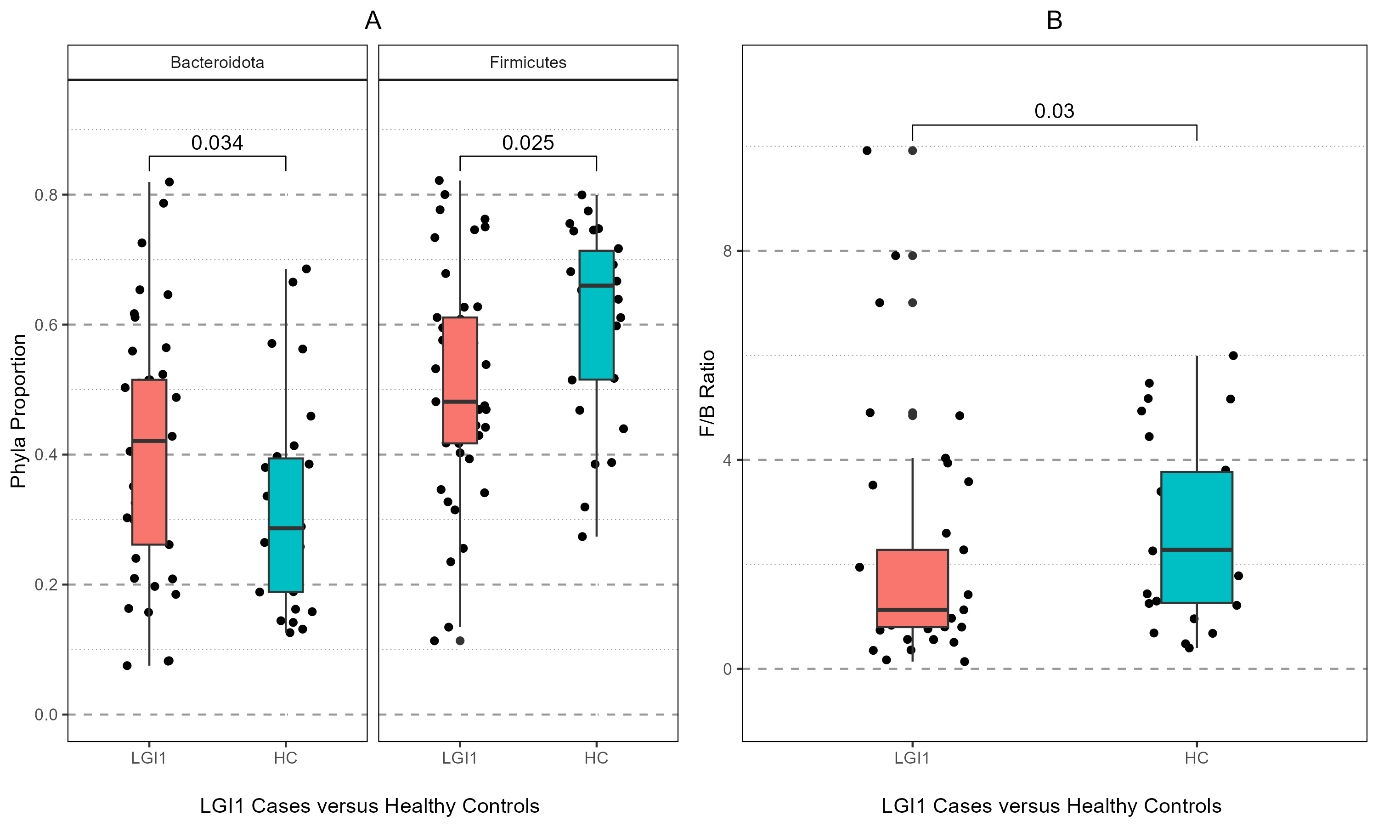


**Supplemental Figure 6 - *Firmicutes*/*Bacteroides* ratio in LGI1-Ab-E cases and healthy controls.** (A) The proportion of the two phyla *Firmicutes* and *Bacteroides* in cases and healthy controls (HC). Comparison bars shown are the p value from Wilcox Signed Rank test. (B) The *Firmicutes*/*Bacteroides* ratio between cases and HC. Individual data points are shown on top of boxplots. Boxplots show the median value, with lower and upper hinges showing the 1st and 3rd quartiles. Whiskers show the largest value no further than 1.5 x the Interquartile Range (IQR) from that range.


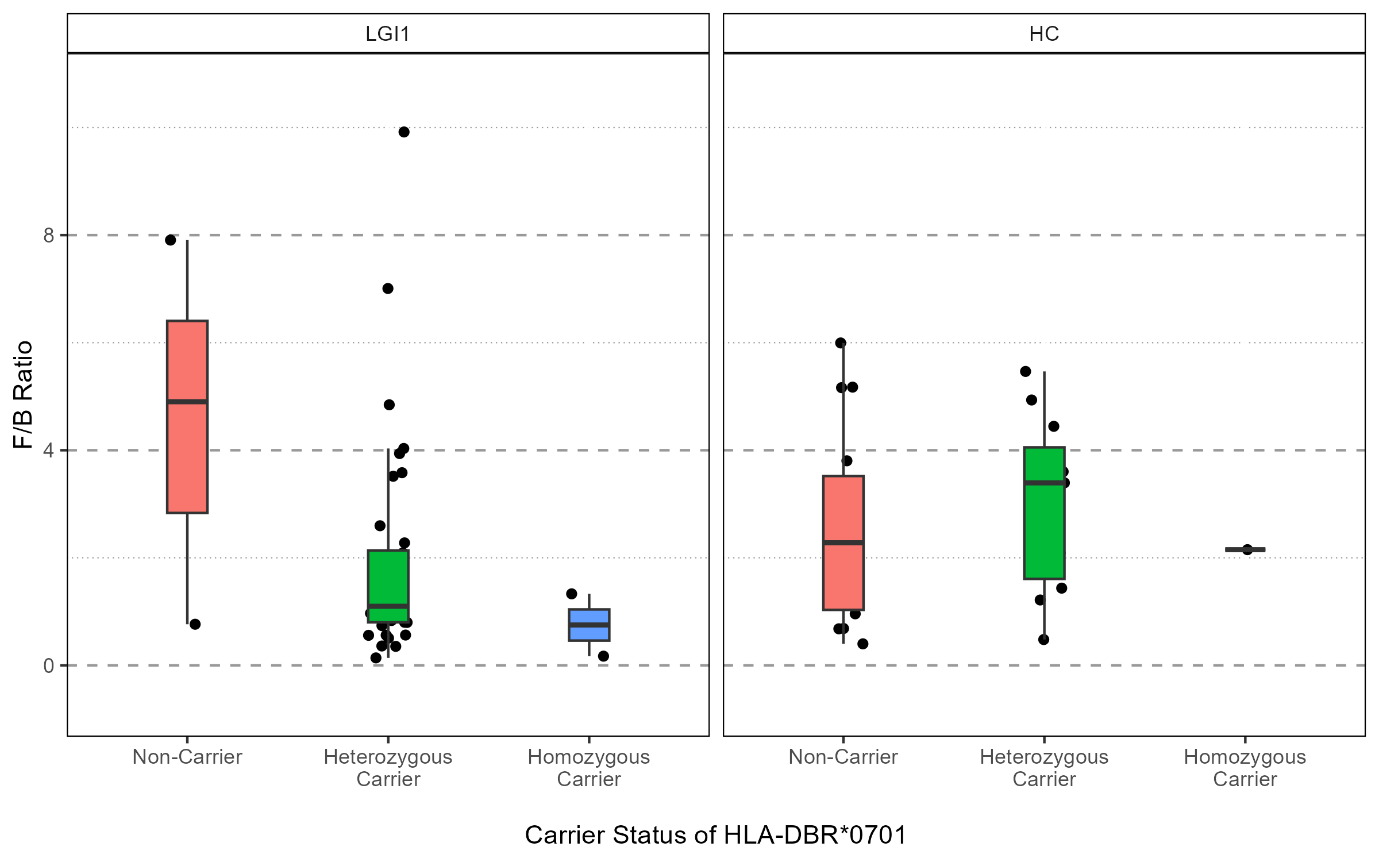


**Supplemental Figure 7 - *Firmicutes*/*Bacteroides* ratio as a function of** **HLA-DBR*0701 dosage.** The ratio of the two phyla *Firmicutes* and *Bacteroides* in LGI1-Ab-E cases and healthy controls (HC) grouped by HLA-DBR*0701 dosage. As HC HLA-DRB1*0701 dosage was imputed from SNP genotypes there is a proportion of missing data. Individual data points are shown on top of boxplots. Boxplots show the median value, with lower and upper hinges showing the 1st and 3rd quartiles. Whiskers show the largest value no further than 1.5 x the Interquartile Range (IQR) from that range.


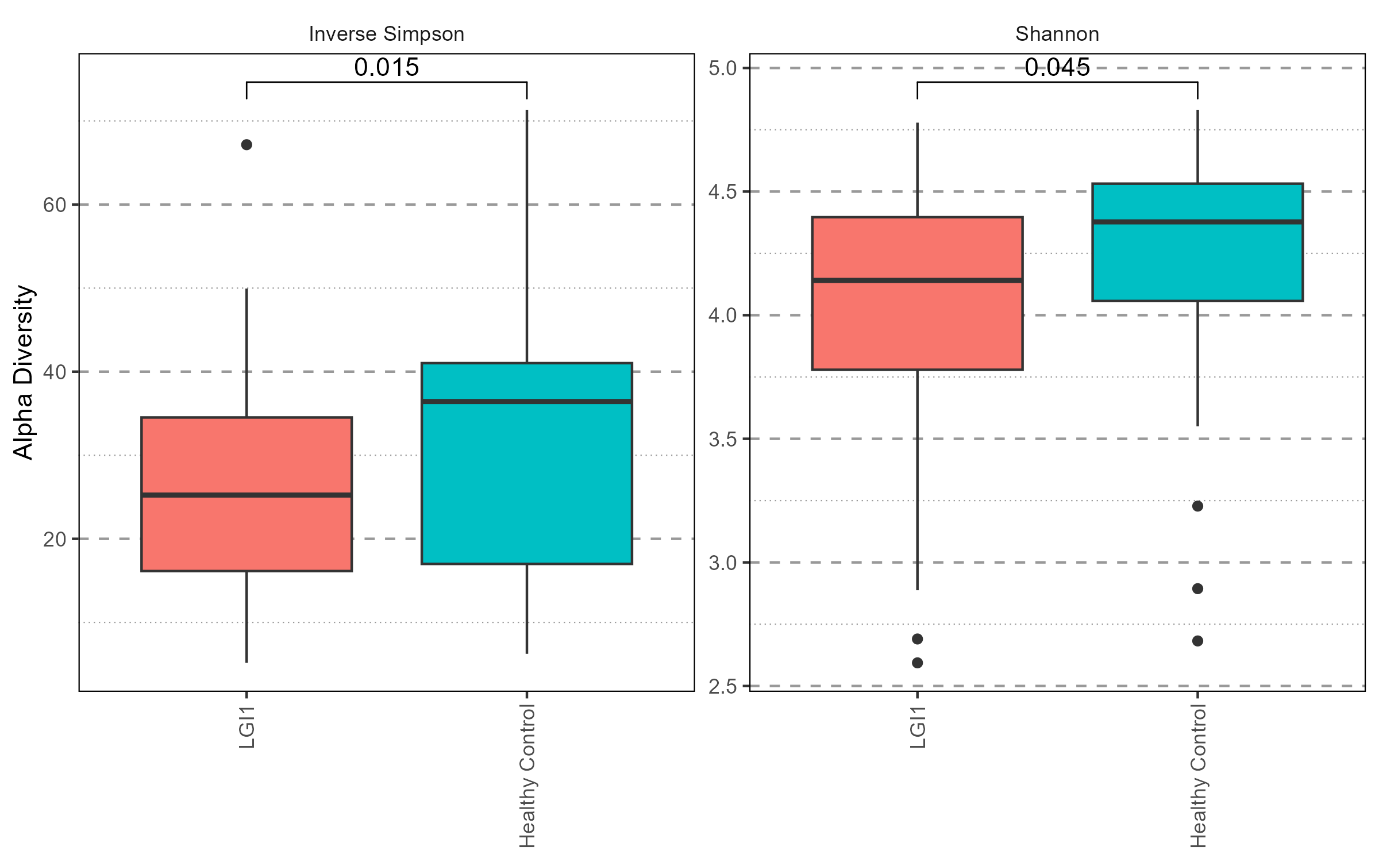


**Supplemental Figure 8 - Measure of Alpha diversity stratified by case type.** Shown are the Inverse Simpson and Shannon alpha diversity measurements. Distributions of alpha diversity values are shown in boxplots. Boxplots show the median value, with lower and upper hinges showing the 1^st^ and 3^rd^ quartiles. Whiskers show the largest value no further than 1.5 x the Interquartile Range (IQR) from that range. Data points beyond these whiskeys are plotted separately as black points.


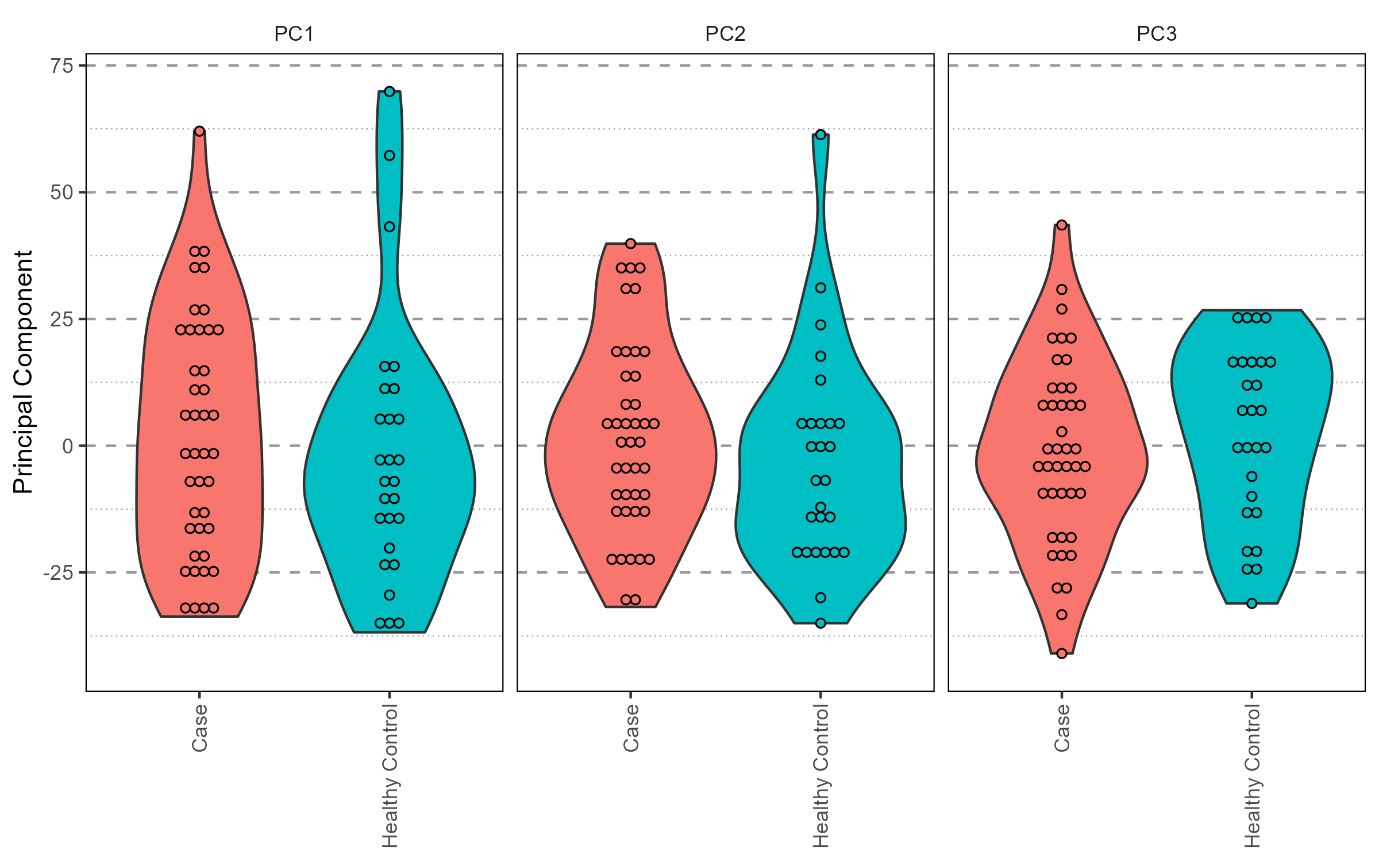


**Supplemental Figure 9 – Distribution of LGI1-Ab-E cases and controls over beta-diversity principal components.** Shown are the sample distributions across the first three Aitchison principal components of beta diversity, with violin plots showing overall distribution. Pairwise comparison values were tested with the Wilcox test of significant differences. All comparison pairs were tested, and p-values adjusted with the Holm method, and not significant differences were found.


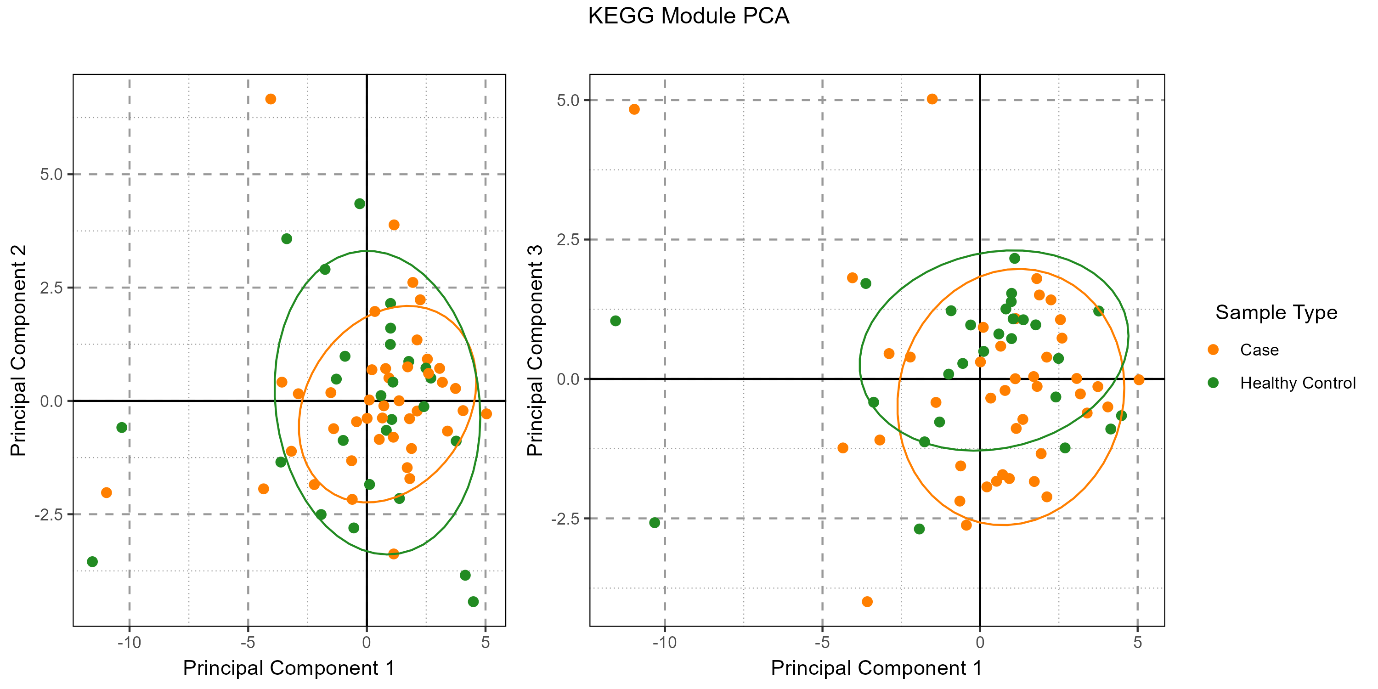


**Supplemental Figure 10 – Aitchison PCA of KEGG Pathway abundance.** LGI1-Ab-E sample case and control type are shown by colour and shape. Ellipses shown are 80% confidence intervals assuming a multivariate t-distribution.


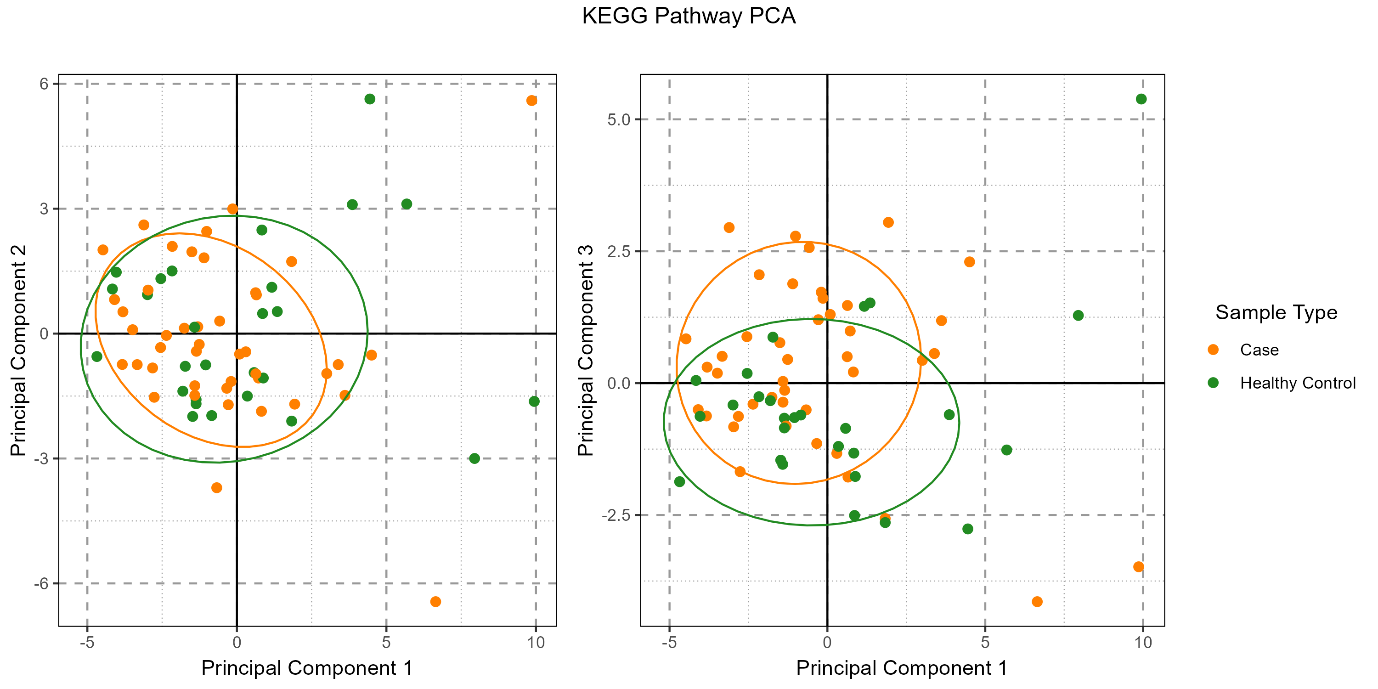


**Supplemental Figure 11 – Aitchison PCA of KEGG Module abundance.** LGI1-Ab-E sample case and control type are shown by colour and shape. Ellipses shown are 80% confidence intervals assuming a multivariate t-distribution.

# Supplemental Data

**Supplemental Data 1 – Dietary and supplement questionnaire** – A blank copy of the dietary and supplement questionnaire provided to study participants.

**Supplemental Data 2 – Differential Species** – The identified differential species between LGI1-Ab-E cases and healthy controls, showing effect size of difference, and adjusted and unadjusted p-values.

**Supplemental Data 3 - LDA Taxa results** – Results of the linear discriminant analysis of differential taxa between LGI1--Ab-E cases and healthy controls, showing which group the enrichment was observed in, the magnitude and adjusted p-value.

**Supplemental Data 4 – Dietary associations with beta-PCs** – The p-values from PERMANOVA tests of association between beta-diversity-derived principal components and dietary habits.

**Supplemental Data 5 – All KEGG Module results** – The raw results of differential abundance of KEGG modules between LGI1-Ab-E cases and healthy controls, including size of effect, and both corrected and uncorrected p-values.

**Supplemental Data 6 – All KEGG Pathway results** – The raw results of differential abundance of KEGG modules between LGI1-Ab-E cases and healthy controls, including size of effect, and both corrected and uncorrected p-values.

**Supplemental Data 7 – Organism orthologues** – The raw results from *blastp* search of the *LGI1* protein and mapped back to our metagenomic data with *blastx*. Shown are the homologue annotation, taxonomy of sequence, percentage identity with *LGI1*, coverage and mapping e-value.
