# Supplementary material for: The gut microbiome associated with LGI1‐antibody encephalitis
Source: Epilepsia. 2025 Aug 6;66(11):4411–24. doi: 10.1111/epi.18556 (PMC12661261; doi:10.1111/epi.18556)
Supplement: Supplementary file 2 — Data S1. Dietary and supplement questionnaire. A blank copy of the dietary and supplement questionnaire provided to study participants. [file EPI-66-4411-s006.docx]

**Questionnaire (for postage with stool sample)**

**Version 3.2 | 13.09.2019**

Please complete the following short questionnaire and return it with your specimen in the postal box provided. Please DO NOT send us any personal information (e.g. name, address etc.).

**Sample collection**

**1. Date you collected your stool sample:**

**2. Time you collected your stool sample:**

**3. Date you sent the stool sample:**

**4. How often do you pass a bowel movement (stool)?** *Please tick as appropriate*

a. Over 3 times a day b. 1-3 times a day

c. 1-3 times per week d. Less than once a week

**Current medication**

**1. Have you changed any medication use (including over-the-counter medications) since your last visit to the clinic? If yes, please provide details below:**

**2. In the last 6 months have you used antibiotic tablets by mouth?**

a. Yes

b. No

c. Not sure

If yes, what were the antibiotics called?

If yes, what were the antibiotics used for?

If yes, what date did you start the course and what was the duration of the course?

**Diet** *Please tick as appropriate*

**1. Do you have any dietary restrictions (e.g. allergy, intolerances)?**

a. Yes

b. No

If yes, please provide details?

**2. What are your dietary preferences with respect to meat?**

a. Standard diet (includes red meat, poultry and seafood)

b. Standard diet with poultry and seafood (no red meat)

c. Pescetarian (no red meat or poultry)

d. Vegetarian (no meat)

e. Vegan (no meat, dairy or animal products)

**3. Do you regularly take probiotics (yakult etc.)?**

a. Never b. rarely (1-3 times per week)

c. 4-6 times per week d. Daily

If you answered b, c or d please provide the probiotic supplement name: _____________________

**4. In the last week have you consumed products that contain live cultures (e.g. yoghurt, kefir, sauerkraut)**

a. Never b. 1-3 times

c. 4-6 times d. Daily

| **5. In the last week how often did you eat the following food groups?**  *(Please tick the boxes that apply)* | Never | 1-3 times | 4-6 times | Daily | If daily, please give the approximate number of times (e.g. 1) |
| --- | --- | --- | --- | --- | --- |
| Dairy products (e.g. milk, cheese etc.) |  |  |  |  |  |
| Acidic fruits (e.g. oranges, apples, berries) |  |  |  |  |  |
| Non-acidic fruits (e.g. bananas) |  |  |  |  |  |
| Brassica vegetables (includes cauliflower, broccoli, cabbage and brussel sprouts) |  |  |  |  |  |
| Non-brassica vegetables (those not listed above) |  |  |  |  |  |
| Breads, cereals and starches (includes bread, rice, pasta) |  |  |  |  |  |
| Red meat (includes beef, pork and lamb) |  |  |  |  |  |
| White meat (e.g. chicken, turkey etc.) |  |  |  |  |  |
| Fish |  |  |  |  |  |
| Eggs |  |  |  |  |  |
| Simple sugars in processed foods (includes chocolate, sweets, fruit juice, fizzy drinks, sugar in hot drinks etc.) |  |  |  |  |  |
| Products containing caffeine (E.g. tea, energy drinks etc.) |  |  |  |  |  |
| **6. In the last week how often did you take vitamin / other supplements?** |  |  |  |  |  |
| Vitamin B6 |  |  |  |  |  |
| Vitamin B12 |  |  |  |  |  |
| Vitamin C |  |  |  |  |  |
| Vitamin D |  |  |  |  |  |
| Zinc |  |  |  |  |  |
| Magnesium |  |  |  |  |  |
| Selenium |  |  |  |  |  |
| Folate |  |  |  |  |  |
| **7. In the last week have you taken the following?** |  |  |  |  |  |
| Antacids (heart burn tablets) or other stomach acid reducing drugs |  |  |  |  |  |
| Laxatives |  |  |  |  |  |
| Protein supplements |  |  |  |  |  |
| Dietary meal replacements (e.g. dietary shakes) |  |  |  |  |  |

**Further Information.** If you would like more information or have any queries please contact your local study team on the information below:
